# Mulberry Extracts Alleviate A*β*_25–35_-Induced Injury and Change the Gene Expression Profile in PC12 Cells

**DOI:** 10.1155/2014/150617

**Published:** 2014-12-17

**Authors:** Nan Song, Hongpeng Yang, Wei Pang, Zhiwei Qie, Hao Lu, Long Tan, Haiqiang Li, Shoudan Sun, Fuzhi Lian, Chuan Qin, Yugang Jiang

**Affiliations:** ^1^Department of Nutrition, Tianjin Institute of Health and Environmental Medicine, Tianjin 300050, China; ^2^Comparative Medicine Center, Institute of Laboratory Animal Science, Peking Union Medical College (PUMC) and Chinese Academy of Medical Sciences (CAMS), Beijing 100021, China; ^3^Tianjin Agricultural College, 22 Jinjing Highway, Tianjin 300384, China; ^4^Center for Disease Control and Prevention, PLA Chengdu Military Area Command, Chengdu 610021, China; ^5^Department of Preventive Medicine, Hangzhou Normal University, Hangzhou 311121, China

## Abstract

Mulberry, which contained high amounts of anthocyanins, has been used in traditional Chinese medicine. Mulberry fruit extracts (ME) have demonstrated the antioxidant activity and neuroprotection. The study was to investigate the neuroprotective efficacy of ME against *β*-amyloid 25–35- (A*β*_25–35_-) induced PC12 cells injury. Cells preincubated with or without ME (200 *μ*g/mL) for 24 h were treated with A*β*_25–35_ (20 *μ*mol/L) for another 24 h. Cell viability was assessed by MTT, gene expression profiles were examined by cDNA microarrays, and RT-PCR were used to confirm the results of microarray assays. ME pretreatment was found to neutralize the cytotoxicity and prevent A*β*_25–35_-induced cells injury. Analyses of gene expression profile revealed that genes involving cell adhesion, peptidase activity, cytokine activity, ion binding activity, and angiogenesis regulation were significantly modulated by ME pretreatment. Among those genes, Apaf1, Bace2, and Plcb4 were enriched in the “Alzheimer's disease-reference pathway” and downregulated after ME intervention. RT-PCR results showed that ME preincubation could significantly inhibit A*β*_25–35_ increased mRNA levels of these three genes. Overall, ME pretreatment could substantially alleviate PC12 cells injury and downregulate expression of AD-related genes, such as Apaf1, Bace2, and Plcb4. This study has a great nutrigenomics interest and brings new and important light in the field of AD intervention.

## 1. Introduction

Alzheimer's disease (AD) is the most common form of dementia in the elderly. AD, characterized by the progressive degeneration of cognition and memory, is correlated with the appearance of neurofibrillary tangles, senile plaques, and loss of neurons in the brain [1–4]. The processing of amyloid precursor protein (APP), a type I transmembrane glycoprotein, plays an important role in the development of AD [5, 6]. In the amyloidogenic pathway, cleavage of APP by *β*-secretase results in the release of a soluble, 110 kDa N-terminal fragment, sAPP*β*, and a 12 kDa membrane-anchored C-terminal fragment, CTF*β*. Subsequently, CTF*β* is cleaved by *γ*-secretase and generates a 4 kDa A*β* peptide [7]. A*β* peptide is the major component of senile plaques and has been suggested to play a causal role in the development and progression of AD [8]. A*β* can trigger a cascade of pathogenic events such as culminating of neuronal apoptosis/death, dystrophy of neurites, excitoactivation of glutamate receptors, and induction of oxidation stress [9]. A*β*_25–35_, a synthetic peptide corresponding to amino acids 25–35 in A*β*_1–40_ and A*β*_1–42_, possesses the same *β*-sheet structure and exhibits large *β*-sheet fibrils [10, 11]. It retains most physical and biological properties of full length-A*β*, including its toxicity [12]. More importantly, A*β*_25–35_ is a particularly intractable peptide because it aggregates rapidly, unlike the full length-A*β*, which requires aging for more than 1 week before it aggregates and becomes toxic [13]. As such, it is often used for the* in vitro* study.

Anthocyanins are a group of naturally occurring phenolic compounds that are responsible for the brilliant color of blue, red, and purple of leaves, flowers, and fruits [14]. Because of significant property of anthocyanin is antioxidant activity, the neuroprotective effect of anthocyanin has received a lot of attention in the field of nutrition research [4, 15]. Many studies showed that effects of the antioxidant activity and neuroprotection of anthocyanins* in vitro*. At the same time,* in vivo* test also confirmed that anthocyanins can reduce the injury area of cerebral ischemic damage in rat [16, 17]. Mulberries (*Morus alba*) have been used in traditional oriental medicine throughout world and in particular in China and contain high amounts of anthocyanins [14, 17, 18]. Zou et al. optimized the microwave-assisted extraction (MAE) conditions of anthocyanins from mulberry using response surface methodology (RSM). Under these conditions, 54.72 mg anthocyanins were obtained from 1.0 g mulberry powder. Furthermore, 8 anthocyanins were identified by high-performance liquid chromatography-electrospray ionization-mass spectrometry (HPLC-ESI-MS) in mulberry extract. Among them cyanidin-3-glucoside and cyanidin-3-rutinoside are the major anthocyanins in mulberry [19, 20]. Studies showed that black-colored mulberry fruit extracts contain the highest levels of anthocyanin, total phenolic, and flavonoid as well as strongest antioxidant compared with other colors of mulberry fruit extracts [8, 21]. Mulberry fruits exhibit a variety of biological and physiological effects, such as antithrombotic, antioxidant, antimicrobial activity, anti-inflammation, and neuroprotection [4]. In recent years, many papers have been published on the neuroprotective effects of mulberry extracts (ME). Animal studies found that mulberry fruits and their neuroprotective constituent—cyanidin-3-*O*-*β*-D-glucopyranoside (C3G), isolated from the mulberry fruits, can alleviate the cerebral ischemic injury and aging-associated neuronal damage* in vivo* using a mouse-brain-injury model with a transient middle cerebral artery occlusion (MCAO) [10]. Shih et al. (2010) found that ME, which are rich in phenolics and anthocyanins increased the antioxidant enzymes activities (Glutathione peroxidase, Catalase) and improved learning and memory ability in senescence-accelerated mice (SAMP) [4]. In* in vitro* experiments, anthocyanins in ME can inhibit A*β*_25–35_ spontaneous aggregation into oligomers and their neurotoxicity in human neuronal SH-SY5Y cells and have neuroprotective effects on the PC12 cells exposed to hydrogen peroxide and oxygen glucose deprivation (OGD) [14, 19].

Yet few studies have used PC12 cells as A*β*_25–35_-induced injury model to investigate cytoprotective and neuroprotective effects of ME* in vitro*. Furthermore, mechanisms that ME pretreatment might inhibit development of AD have not been elucidated clearly. To explore mechanisms involved, we use A*β*_25–35_ treated PC12 cell as an* in vitro* model to investigate the role of ME and use the genomic techniques to quickly and accurately quantify vast numbers of potential gene expression changes after ME pretreatment. This study could thus have a great nutrigenomics interest and bring new and important light in the field of Alzheimer's disease intervention.

## 2. Materials and Methods

### 2.1. Preparation of Black Mulberry Extracts

Mature mulberry fruits (*Morus nigra L.*) were harvested from a local orchard in turfan depression, Xinjiang Uygur Autonomous Region in China, and purchased from Xinjiang Bencaotang Traditional Chinese Herbal Decoction Pieces Co. Ltd. (Lot NO.: 10121805, Tel.: +86 991 4639388). Mulberry extracts (ME), without any of the amino acids and vitamins, were prepared in the laboratory as described below. Blended fresh mulberry fruits were extracted with 60% alcohol and dehydrated in a freeze dryer (FD-I, USA) for 48 h in a vacuum freezer (−50°C). Total anthocyanin content in black mulberry extracts was 6.8% (0.068 mg/mL) as measured using a full wavelength UV spectrophotometer scanning (wavelength 282 nm). ME stock solution was prepared by dissolving ME in Dulbecco's modified Eagle's medium (DMEM, Gibco, USA) (20 mg/mL) and sterilized through a 0.22 *μ*m filter. At the time of treatment, ME stock solution was further diluted with culture medium to a concentration of 200 *μ*g/mL.

### 2.2. Cell Culture and Treatment

PC12 cell line (rat adrenal pheochromocytoma, The Shanghai Institute of Biochemistry and Cell Biology, SIBCB, China) was grown in high glucose DMEM medium supplemented with 5% (v/v) fetal bovine serum (FBS, HyClone, USA), 10% heat-inactivated horse serum (HS, HyClone, USA), and 1% penicillin/streptomycin (Sigma, USA) in a 5% CO_2_ incubator at 37°C and saturated humidity. To induce neuronal differentiation, cells were treated with 50 ng/mL of nerve growth factor (NGF, Peprotech, USA). When cells were at about 80% confluence, the medium was replaced with DMEM medium containing 1% FBS, 1% penicillin, and 1% streptomycin with NGF (50 ng/mL). At the treatment, cells were divided into four groups: (1) control group: no treatment, (2) A*β*_25–35_ group: cells were treated with 20 *μ*mol/L A*β*_25–35_ for 24 h, (3) ME plus A*β*_25–35_ group: cells were pretreated with 200 *μ*g/mL ME for 24 h, and then the medium was discarded and switched to that containing 20 *μ*mol/L A*β*_25–35_ for another 24 h, and (4) ME group: cells were treated with 200 *μ*g/mL ME alone for 24 h [22]. Before the experiment, the A*β*_25–35_ peptide solution was incubated at 37°C for a week to produce the conformation of fibril or aggregation before adding to PC12 cells.

### 2.3. Cell Viability

Cell viability was determined by MTT assay. Briefly, PC12 cells were plated at a density of 1 × 10^4^ cells/well in 96-well plates and treated with ME, A*β*_25–35_, or ME plus A*β*_25–35_ at indicated concentration, respectively. At the time of assay, cells were washed with PBS and incubated with MTT reagent (5 g/L, 10% v/v, Sigma, USA) at 37°C for 4 hours. The resulting MTT formazan crystals were solubilized by dimethylsulfoxide (DMSO, 150 *μ*L) for 10 min at room temperature. Optical density was measured at 570 nm using a microtiter plate reader (uQuant). Results were expressed as the percentage of MTT reduction as compared with control group.

### 2.4. RNA Isolation and cDNA Synthesis

Total RNA was extracted from various groups using Trizol reagent (Invitrogen, Life Technologies, USA.) following instruction. The extracted RNA was further purified using RNeasy columns (QIAGEN RNeasy Mini Kit, Germany). The quantity and quality of the RNA was determined by Abs260/Abs280 ratio (≥1.80), Abs260/Abs230 ratio (≥1.50), and ethidium bromide fluorescence of RNA resolved in 1% agarose gels. cDNA was synthesized using an oligo dT-T7 promoter primer (Roche Molecular Biochemicals, Mannheim, Germany) and used as a template for* in vitro* transcription.

### 2.5. Microarray Analysis

Fluorescently labeled probes for oligo microarray analysis were prepared using Amino allyl Message Amp aRNA kit (Applied Biosystems, Foster City, CA, USA) as instructed. Labeled probes were hybridized to a Phalanx Rat OneArray containing 24358 rat specific probe sets (Phalanx Biotech Group, Inc., China Taiwan) at 50°C for 16 hrs. Slides were washed with 2 × SSC/0.2% SDS at 42°C for 5 min, 2 × SSC at 42°C for 5 min, 2 × SSC at 25°C for 5 min, and then ten times with 0.2 × SSC. Then spin dried slides were scanned using an Affymetrix Gene Array scanner (USA) and analyzed with GenePix 3.0 software (Axon Instruments, Union City, CA) to obtain gene expression ratios. Logged gene expression ratios were normalized by Lowess regression [23]. Rosetta Resolver System (Rosetta Biosoftware) was used for data preprocess and differential gene expression analysis. Cluster 3.0 and Tree View (http://rana.lbl.gov/EisenSoftware.htm) were employed to clustering analysis.

### 2.6. Reverse Transcription Polymerase Chain Reaction (RT-PCR)

Total RNA was prepared as mentioned above using the same samples. PrimeScript RT Master Mix Perfect Real Time kit (Takara) was used to synthesize first strand cDNA as described by the manufacturer. Specific DNA sequences were amplified with a PCR mixture (TIANGEN Biotech, China) and resolved on a 2% agarose gel. PCR primers were indicated as follows: Apaf 1, 5′-ATGTTATCCCTGTGGAGAG- TGG-3′ (sense) and 5′-CACCAACTAAAGACACGACGAG-3′ (antisense); Bace 2, 5′-TTGTGGACACCGGAAGCAGTAA-3′ (sense) and 5′-CCTCAAAGCCCTTGGAGTGGTA-3′ (antisense); Plcb 4, 5′-GCCCATTACTTCATCAGTTCCT-3′ (sense), 5′-TACACATTGCTTTTCCGTGAGT-3′ (antisense); *β*-actin, 5′-CACCCGCGAGTACAACC TTC-3′ (sense) and 5′-CCCATACCCACCATCACACC-3′ (antisense).

### 2.7. Statistics

Data are expressed as the mean ± standard deviation. Statistical analysis was performed by one-way analysis of variance (ANOVA) and post hoc Bonferroni/Dunn test. Values of *P* less than 0.05 were considered statistically significant.

## 3. Results

### 3.1. Effect of ME on Viability in A*β*_25–35_ Treated PC12 Cells

**Figure 1:**
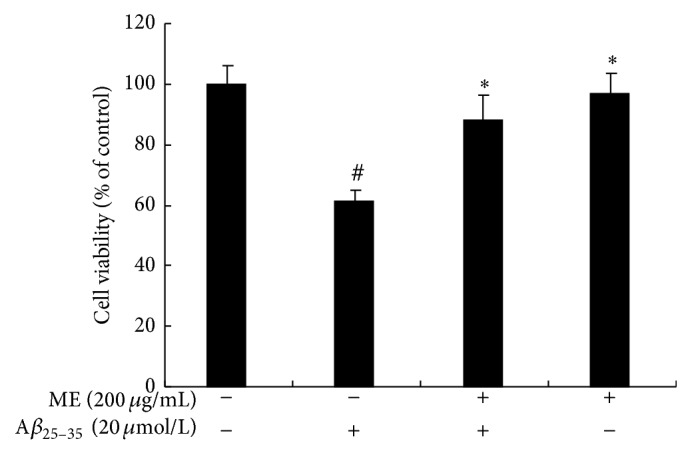
Cytoprotective effects of mulberry extracts in A*β*_25–35_-induced PC12 cells. PC12 cells were pretreated with or without ME (200 *μ*g/mL) for 24 h and exposed to A*β*_25–35_ (20 *μ*mol/L) for 24 h. The cytotoxicity was measured by MTT assay. The viability of the untreated cells was set to 100%. The values represent mean (%) ±S.D. of each group (*n* = 8) of three different cultures. (#) Significantly different from the control group (*P* < 0.05). (∗) Significantly different from the A*β*_25–35_ group (*P* < 0.05).

A*β*_25–35_ treatment resulted in a significant decrease [(38.3 ± 6.9)%] in cell viability in PC12 cells as compared with the control group (*P* < 0.05, *n* = 8). ME treatment alone did not significantly affect cell viability; ME pretreatment inhibited A*β*_25–35_-induced cell death by (29.8 ± 8.7)% (Figure 1). The result shows that ME attenuated A*β*_25–35_-induced cell injury in PC12 cells.

### 3.2. Effect of ME on Gene Expression Profile of A*β*_25–35_ Treated PC12 Cells

Gene expression profile was analyzed by microarray method using 2-fold change (*P* < 0.05) as cut point. Of the 24358 genes on the chip, the expression levels of 5 genes (0.02%) were increased and those of 16 genes (0.07%) were decreased in cells treated with A*β*_25–35_ as compared with control. Pretreatment with ME resulted in increased expression of 55 genes (0.2%) and decreased expression of 98 genes (0.4%) as compared with A*β*_25–35_ group. Changes in gene expression reflect the influence of ME-supplement on the cells (Table 1).

### 3.3. Cluster Analysis and Gene Ontology (GO) Classification

**Figure 2:**
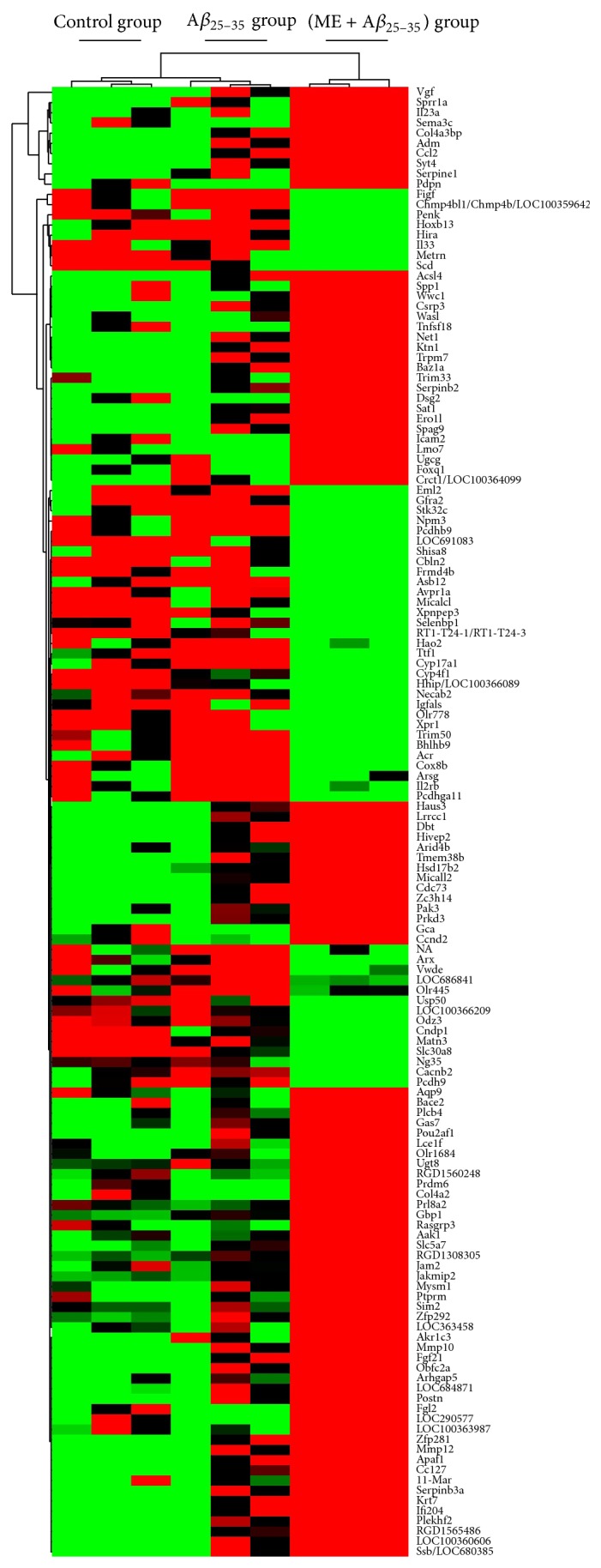
Hierarchical clustering of PC12 cells analyzed with the microarray chip. PC12 cells were pretreated with or without ME for 24 h and exposed to A*β*_25–35_ (20 *μ*mol/L) for 24 h. Data are representative of three different experiments. Up- and downregulated genes are represented in red and green colors, respectively.

**Figure 3:**
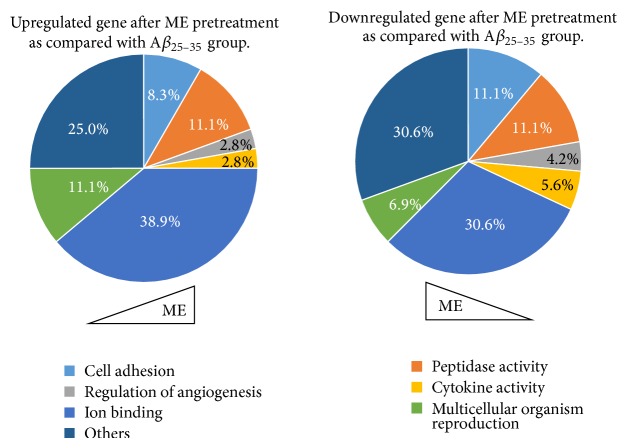
The results of GO classification in ME pretreatment group.

For advanced data analysis, all biological replicates were pooled and calculated to identify differentially expressed genes based on the threshold of fold change and *P* value. The correlation of expression profiles between biological replicates and treatment conditions was demonstrated by unsupervised hierarchical clustering analysis. For this microarray project, the number of genes clustered was 144 (Figure 2). According to biological process ontology analysis, GO classification items enriched in the difference-expression genes were mainly related to cell adhesion, peptidase activity, cytokine activity, ion binding activity, and angiogenesis regulation in ME pretreatment group (*P* < 0.05) (Table 2, Figure 3).

### 3.4. Pathway Analysis and Verification

**Figure 4:**
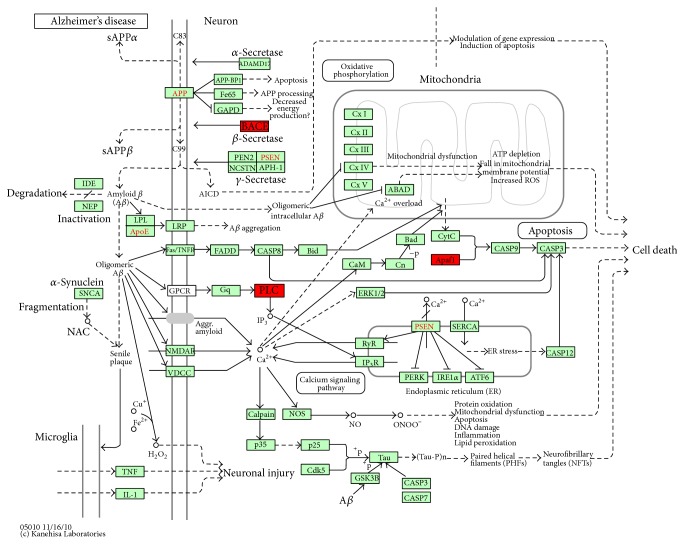
Screened differentially expressed genes were rich in Alzheimer's disease-reference pathway (http://www.genome.jp/kegg-bin/show_pathway?rno05010).* Note.* The gene in red box represents downregulated expression after ME pretreatment in Alzheimer's disease-reference pathway.

**Figure 5:**
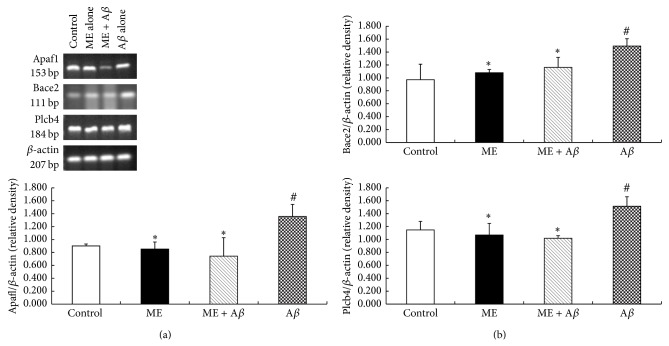
mRNA expression of Apaf1, Bace2, and Plcb4 genes measured by RT-PCR. Cells were pretreated with or without ME for 24h and exposed to A*β*_25–35_ (20 *μ*mol/L) for 24 h. (a) Typical mRNA bands of Apaf1, Bace2, Plcb4 and *β*-actin from control group, 200 *μ*g/mL ME treatment alone group, pretreatment group with 200 *μ*g/ml ME, and treatment group with A*β*_25–35_ alone. (b) Apaf1, Bace2 and Plcb4 mRNA levels were normalized to *β*-actin mRNA level and presented as relative value. The values represent mean ± S.D. of the each group (*n* = 6) of three independent experiments. (#) Significantly different from the control group (*P* < 0.05). (∗) Significantly different from the A*β*_25–35_ group (*P* < 0.05).

Based on NCBI database, the screened differentially expressed genes Apaf1, Bace2, and Plcb4 were enriched in the “Alzheimer's disease-reference pathway” (*P* < 0.01) (Figure 4) and meanwhile significantly downregulated in A*β*_25–35_-injured PC12 cells after ME intervention as compared with A*β*_25–35_ group (*P* < 0.05) (Table 3). RT-PCR method was used to verify the changes of Apaf1, Bace2, and Plcb4 mRNA expression.  Figure 5 showed that mRNA levels of Apaf1, Bace2, and Plcb4 were upregulated in A*β*_25–35_ treated PC12 cells as compared with control group (*P* < 0.05), increased by (82.6 ± 21.1)%, (53.5 ± 13.0)%, and (31.9% ± 1.3)%, respectively, while 200 *μ*g/mL ME preincubation for 24 h significantly inhibited A*β*_25–35_-induced upregulation of Apaf1, Bace2, and Plcb4 mRNA expression in PC12 cells (*P* < 0.05), decreased by (61.5 ± 13.2)%, (33.9 ± 4.3)%, and (43.1 ± 9.3)%, respectively, which was consistent with the microarray analysis. These results indicated that ME pretreatment could substantially downregulate Apaf1, Bace2, and Plcb4 mRNA expression levels in A*β*_25–35_-injured PC12 cells.

## 4. Discussion

There is an increasing interest in the beneficial effects of nutritional antioxidants on health via the delay of aging and age-related diseases [24–29]. The observed protection may be the result of the antioxidant and anti-inflammatory properties of the polyphenolic compounds found in these fruits and vegetables [30]. Our previous studies found that pretreatment of PC12 cells with 200 *μ*g/mL ME could almost completely reverse A*β*_25–35_-induced neuronal injury, counteract ROS formation, and inhibit apoptosis. The results suggested that ME could alleviate A*β*_25–35_-induced injury in PC12 cells, which might be associated with the antioxidative and antiapoptosis effects [22]. In this study, we further investigate the possible mechanisms involved.

To explore the molecular mechanisms of neuroprotective effect of ME, the transcription of 24,358 genes was analyzed by gene chips. Combined with bioinformatics analysis, the gene expression profiles in samples were significantly affected. Further analysis shows that in ME pretreatment group, the downregulated genes were mainly related to cell adhesion, cytokine activity, and angiogenesis regulation, and upregulated genes were mostly related to ion binding activity and multicellular organism reproduction. Based on NCBI database, the screened differentially expressed genes Apaf1, Bace2, and Plcb4 were enriched in the “Alzheimer's disease-reference pathway.” That is, these genes not only play an important role in the development of AD, but also their genes expression products were involved in apoptosis, A*β* formation, and cell or organelle membrane damage. We further validate that preincubation with ME significantly downregulated mRNA levels of Apaf1, Bace2, and Plcb4 in PC12 cells, suggesting that the bioactive components in ME can significantly inhibit cell apoptosis, A*β* formation, or membrane damage-associated gene expression.

Apoptotic protease-activating factor 1 (Apaf1), a tumor suppressor gene, is essential for regulation of mammalian development and induction of cell apoptosis [31–33]. The core of the mitochondria-dependent pathway is “apoptosome,” comprised by Apaf1, cytochrome C (Cyt.C), and dATP/ATP [34]. Furthermore, a series of apoptosis transduction cascade reactions* in vivo* act on Apaf1 firstly and then regulate apoptosome further [35]. In addition, Apaf1 is high expression in the peripheral blood leukocytes, spleen, fetal lung, kidney, and brain [36]. Changes in protein levels of Apaf1 determine cell proliferation or apoptosis. So the abnormal expression and function of Apaf1 is associated with the development of many human diseases [37]. The previous result found that ME preincubated cells can effectively reduce the rate of cell apoptosis [22]. The gene chip results and further validation found that A*β*_25–35_ increased expression of Apaf1 and ME pretreatment reversed the upregulation of Apaf1 expression induced by A*β*_25–35_. That is, ME could alleviate the apoptosis by the way of suppressing gene expression of Apaf1 in PC12 cells. It is further confirmed that Apaf1 is essential for the normal development of the brain [38] and the changes of transcription level of which may associate with the development of AD.

*β*-Secretase (*β*-site APP cleaving enzyme, BACE) is the rate limiting enzymatic activity in the production of the amyloid-*β* peptide (A*β*) and is thought to be involved in Alzheimer's disease (AD) pathogenesis [39]. Though Bace2, the homologue of Bace1, is also expressed in the brain, its potential role in AD has not been clarified completely. However, Bace2 (Asp1, DRAP) also exhibits *β*-secretase-like activity [40, 41]. Transient expression of Bace2 in APP-expressing cells results in an increase in the levels of *β*-secretase derived cleavage products, sAPP*β* and CTF*β*, whose role has dispute in the pathogenesis of AD [7, 42]. So Bace2 maybe play critical role in the development of AD, because accumulation and deposition of A*β* fragments in brain induce A*β*-generated cascade process, which is an important step of AD pathologic processes. That is, by the way of inhibiting BACE2 activity, could directly decrease the level of A*β*. According to microarray analysis and PCR results, Bace2 mRNA in PC12 cells were obviously more overexpressed in A*β*_25–35_ group than those in the control group, while 200 *μ*g/mL ME preincubated for 24 h could significantly inhibit A*β*_25–35_-induced upregulation of Bace2 mRNA expression. Therefore, we speculate that BACE2, not BACE1, as another therapeutic target, is expected to become a promising way to treat AD.

Translation products of Plcb4 gene are PLC-*β* 4, one of the isozymes of phospholipase C-*β* (PLC-*β*). Phospholipase C (PLC), as an important enzyme, is widespread in various cells, having species and cell specificity in the basic biochemical characteristics, function, and subcellular distribution [43, 44]. PLC is the phospholipid component of cellular membrane and also participates in the apoptosis signal transduction in various cells, such as neurons [45, 46]. Other studies have found that PLC is involved in the regulation of oxidative stress caused by oxidative glutamate toxicity induced neuronal cell death in immature cortical neurons and hippocampal neurons [47]. Our previous experimental results and other literature [20] found that ME pretreatment alleviated the damage of membrane structure of cells or organelles, especially preserved the mitochondrial membrane integrity and inhibited the decrease of mitochondrial membrane potential (see supplementary Figure in supplementary materials available online at http://dx.doi.org/10.1155/2014/150617). In the present research, we noted that Plcb4 mRNA in PC12 cells were obviously more overexpressed in A*β*_25–35_ group than those in the control group, which can be inhibited by ME preincubation. The results indicated that ME could inhibit apoptosis by the way of suppressing gene expression of Plcb4 in PC12 cells.

## 5. Conclusions

In summary, the results suggested that ME pretreatment could substantially alleviate the cell injury induced by A*β*_25–35_, which may be related to the antioxidative and antiapoptotic properties of ME (or anthocyanins). ME may negatively regulate the expression of Apaf1, Bace2, and Plcb4 genes, thereby delaying the development of AD.

## Supplementary Material

Mulberry fruit extracts (ME) contain high amounts of anthocyanins. The supplementary material involved in the preparation processes of mulberry extracts and determination of total anthocyanins content. Mulberries and their major neuroprotective compound—C3G (cyanidin-3-O-*β*-D-glucopyranoside) have demonstrated the neuroprotective effect on a cerebral infarction in mouse brain injury model and H_2_O_2_-induce oxidative damage in PC12 cells. Yet few studies have used mulberry fruit extracts (mixture) as the only intervention substance to investigate cytoprotective and neuroprotective effects on A*β*_25-35_-induced injury model in PC12 cells. So this supplementary material also involved in the morphological shapes of PC12 cells induced by NGF and the morphological shapes of differentiated PC12 cells in different treatment groups. To explore mechanisms involved, we use the genomic techniques to quickly and accurately quantify vast numbers of potential gene expressions. Table 5 and 6 in supplementary material illustrated the significantly changed genes in PC12 cells after different treatment.

## Figures and Tables

**Table 1:** Up- (≥2-fold) and down- (≤2-fold) regulation in gene expression in PC12 cells after exposure to A*β*_25–35_ or pretreatment with ME for 24 h.

Comparison	Downregulated	Upregulated
A*β*_25–35_ versus control	16	5
(ME + A*β*_25–35_) versus A*β*_25–35_	98	55

**Table 2:** Statistical results of GO classification in ME pretreatment group.

Term	Count	Up	Upregulated	Down	Downregulated
Cell adhesion	11	3	Pcdhb9, Igfals, Pcdh9	8	Ptprm, Dsg2, Pdpn, Trpm7, Icam2, Lmo7, Postn, Spp1
Peptidase activity	12	4	Acr, Eml2, Cndp1, Xpnpep3	8	Mmp10, **Bace2**, Serpine1, Serpinb2, Fgl2, **Apaf1**, Mysm1, Mmp12
Regulation of angiogenesis	4	1	Figf	3	Col4a2, Ptprm, Serpine1
Cytokine activity	5	1	Il33	4	Il23a, Ccl2, Ccl27, Spp1
Ion binding	36	14	Cndp1, Pcdhb9, Trim50, Cacnb2, Necab2, Vwde, Scd, Tmem38b, Ng35, Cyp17a1, Pcdh9, Arsg, Xpnpep3, Cyp4f1	22	Syt4, Lmo7, **Plcb4**, Pak3, Acsl4, Zc3h14, Micall2, Trpm7, Csrp3, March11, Mmp12, Tmem38b, Mmp10, Plekhf2, Baz1a, Trim33, Dsg2, Prdm6, Hivep2, Zfp281, Slc5a7, Prkd3
Multicellular organism reproduction	9	4	Acr, Avpr1a, Micalcl, Pcdhga11	5	Ccl2, Ccnd2, Acsl4, Vgf, Prl8a2
Others	31	9	Matn3, Igfals, Il33, Metrn, Figf, Avpr1a, Slc30a8, Scd, Penk	22	Col4A2, Ccl2, Loc363458, Postn, Vgf, Mmp12, Mmp10, L23a, Adm, Wasl, Serpinb2, Sema3c, Spp1, Ccl27, Serpine1, Prl8a2, Ptprm, Pdpn, Trpm7, Gas7, Col4a3bp, Serpin

Total	108	36		72	

*Note*. Upregulated means upregulated genes, Downregulated means downregulated genes.

**Table 3:** Significant genes based on microarray data in AD pathway.

Gene ID	Gene symbol	Gene description	log_2_ (ratio)	*P* value
78963	Apaf1	Apoptotic peptidase activating factor 1	−1.0464	0.00267
288227	Bace2	Beta-site APP-cleaving enzyme 2	−1.0448	0.00001
25031	Plcb4	Phospholipase c, beta 4	−1.0673	0.00381
