# Supplementary material for: Mulberry Extracts Alleviate Aβ 25–35-Induced Injury and Change the Gene Expression Profile in PC12 Cells
Source: Evid Based Complement Alternat Med. 2014 Dec 17;2014:150617. doi: 10.1155/2014/150617 (PMC4280656; doi:10.1155/2014/150617)
Supplement: Supplementary file 1 — Mulberry fruit extracts (ME) contain high amounts of anthocyanins. The supplementary material involved in the preparation processes of mulberry extracts and determination of total anthocyanins content. Mulberries and their major neuroprotective compound—C3G (cyanidin-3-O-β-D-glucopyranoside) have demonstrated the neuroprotective effect on a cerebral infarction in mouse brain injury model and H2O2-induce oxidative damage in PC12 cells. Yet few studies have used mulberry fruit extracts (mixture) as the only intervention substance to investigate cytoprotective and neuroprotective effects on Aβ 25-35-induced injury model in PC12 cells. So this supplementary material also involved in the morphological shapes of PC12 cells induced by NGF and the morphological shapes of differentiated PC12 cells in different treatment groups. To explore mechanisms involved, we use the genomic techniques to quickly and accurately quantify vast numbers of potential gene expressions. Table 5 and 6 in supplementary material illustrated the significantly changed genes in PC12 cells after different treatment. [file 150617.f1.doc]

1. **Preparation process of mulberry extracts**


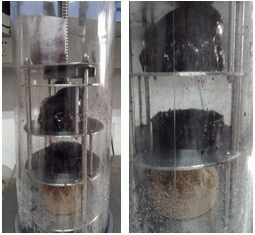


Dried black-colored mulberries

Ethanol (60%) immersion extraction

(Combined and concentrated that 6 days / time , 5 times)

Mulberry initial extracts

Frozen and dried treatment

Mulberry extracts (ME)

Supplementary figure 1 Preparation process of mulberry extracts.

1. **Determination of total anthocyanin in ME (Full Wavelength UV)**


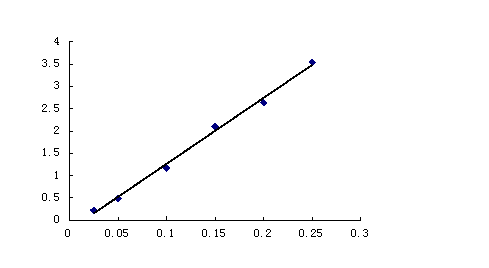


OD value

Concentration of samples（mg/ml）

y=14.789x-0.2127

Supplementary figure 2 The standard curve of samples

The average value of optical density in ME samples is 0.789.

Total anthocyanin in ME is 6.8% (0.068mg/ml).

**3. The morphological shapes of PC12 cells induced by NGF**

**A**

**B**

**C**


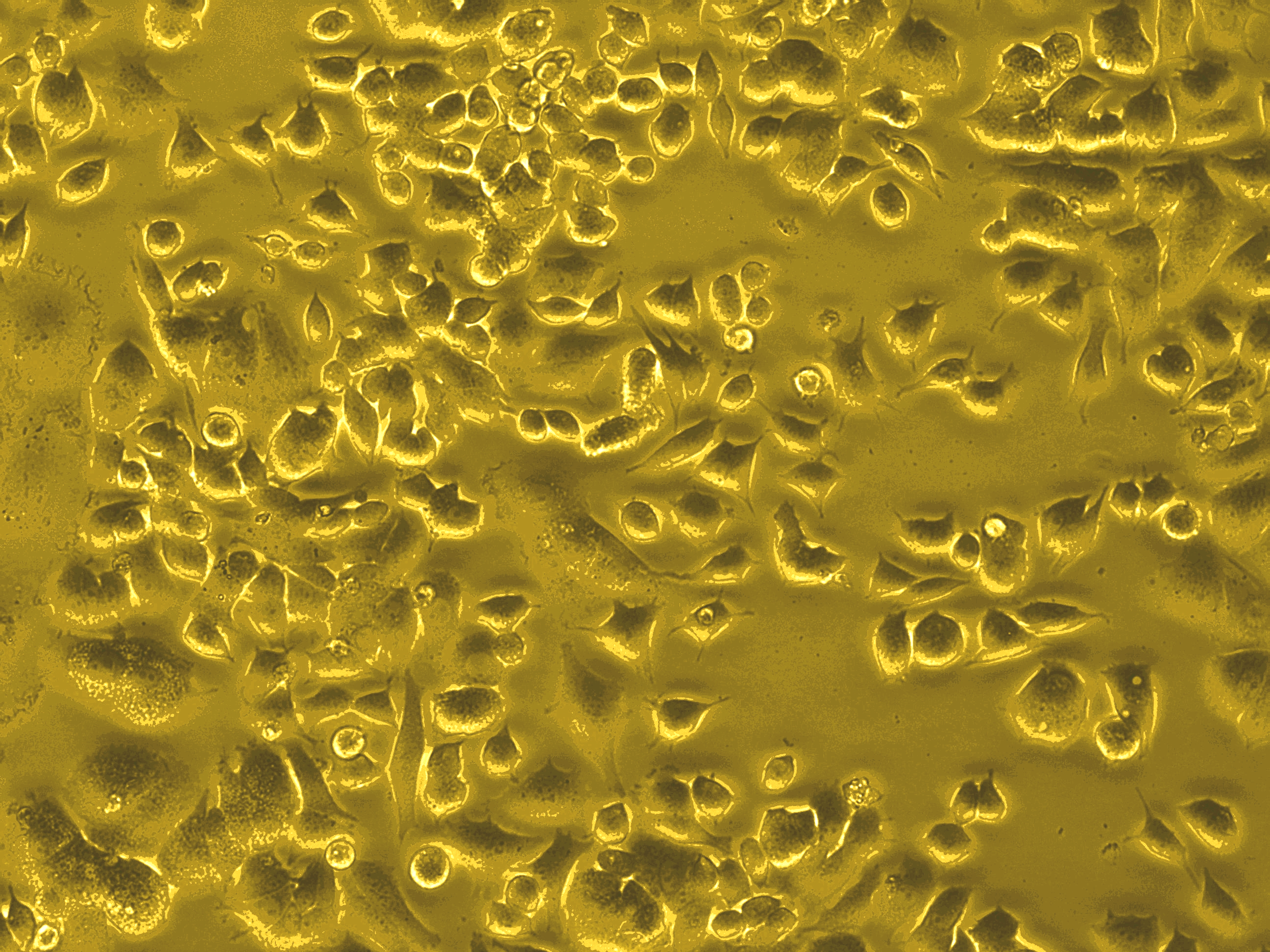

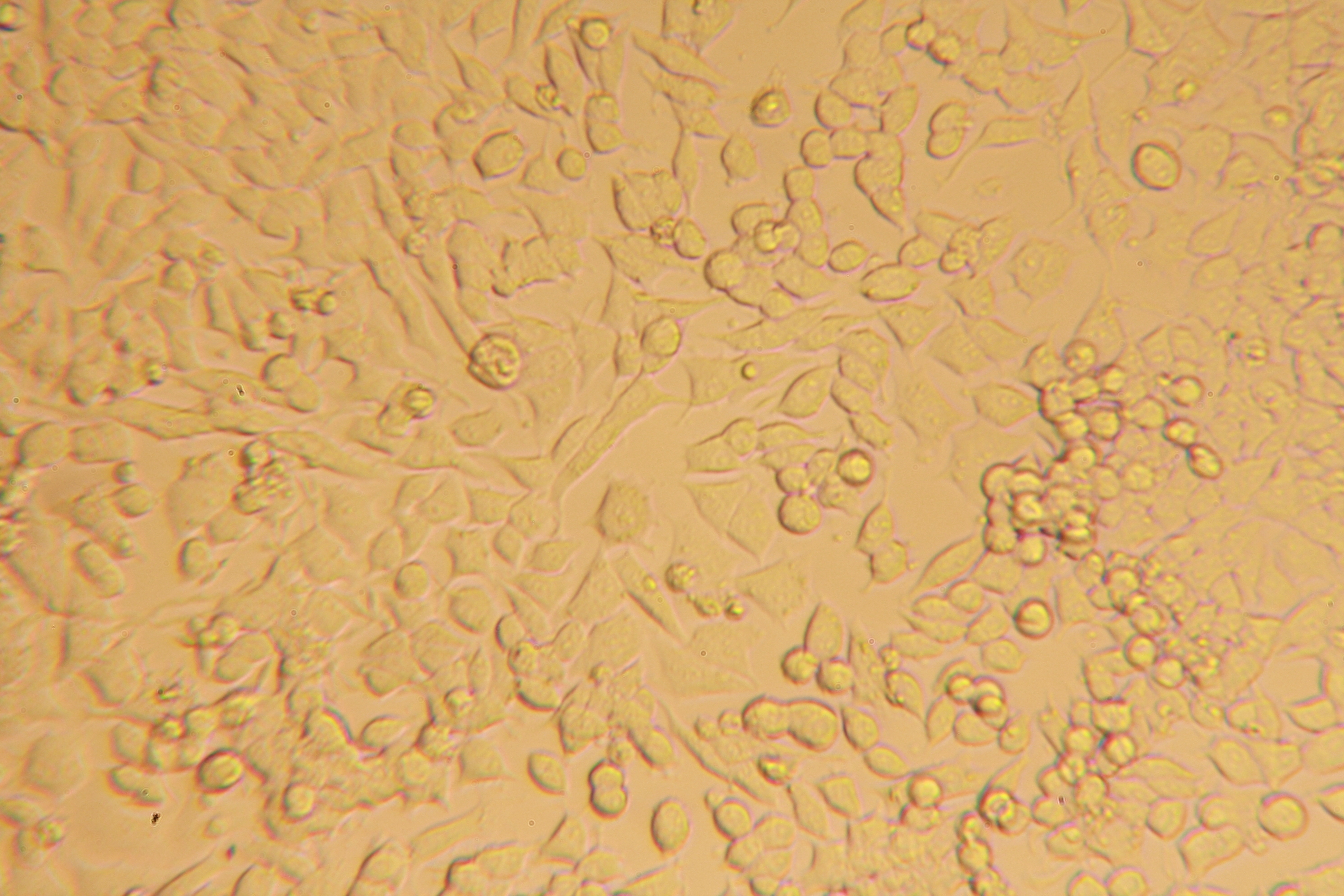

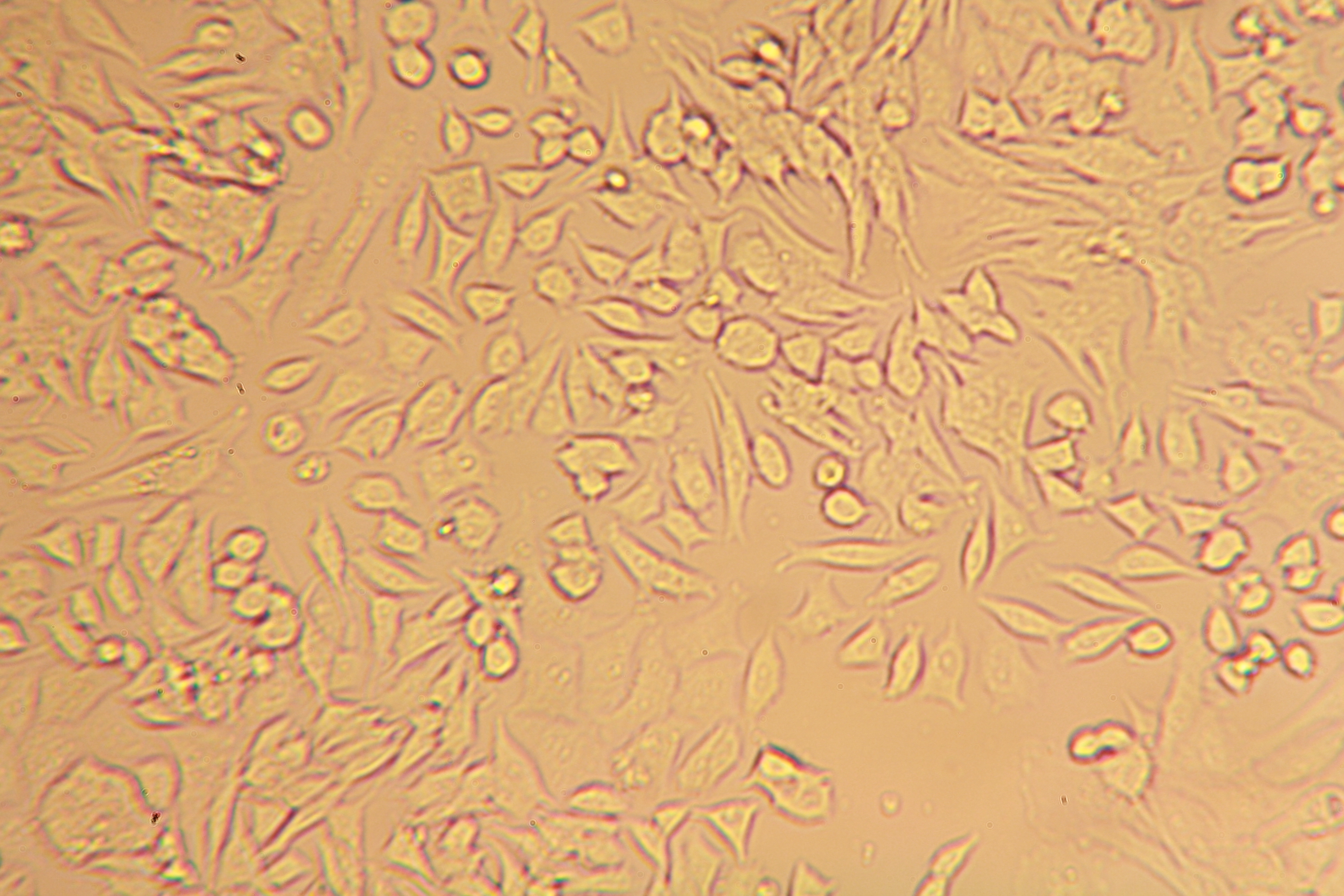


A：PC12 cells cultured in control group; B: PC12 cells cultured in 50ng/ml NGF for 48h; C: PC12 cells cultured in 50ng/ml NGF for 8d

Supplementary figure 3 The morphological shapes of PC12 cells (×200).

**4. Cytoprotective effects of mulberry extrects in Aβ25-35-induced PC12 cell**


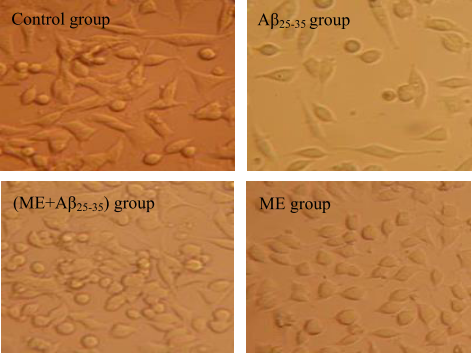


（×200）

Supplementary figure 4 Cytoprotective effects of mulberry extrects in Aβ_25-35_-induced PC12 cells. After pretreating the PC12 cells with ME for 24h, the cells were exposed to Aβ_25-35_ (20μmol/L) for 24h, and the cytotoxicity was measured by MTT assay.

1. **Up-regulated ( ≥ 2.0-fold) and down-regulated ( ≤ 2.0-fold) genes in PC12 cells after ME pretreatment as compared with Aβ_25-35_ group.**

| **Gene ID** | **Gene symbol** | **Gene description** | **Fold change** |
| --- | --- | --- | --- |
| 25146 | Cyp17a1 | Cytochrome P450, family 17, subfamily a, polypeptide 1 | 2.306 |
| 25136 | Gfra2 | GDNF family receptor alpha 2 | 1.930 |
| 84029 | Hao2 | Hydroxyacid oxidase 2 (long chain) | 1.882 |
| NA | NA | NA | 1.880 |
| 287151 | Metrn | Meteorin, glial cell differentiation regulator | 1.701 |
| 365381 | Stk32c | Serine/threonine kinase 32C | 1.692 |
| NA | NA | NA | 1.637 |
| 502731 | Vwde | Von Willebrand factor D and EGF domains | 1.627 |
| 317407 | Bhlhb9 | Basic helix-loop-helix domain containing, class B, 9 | 1.577 |
| 679886|312254|100359642 | Chmp4bl1|Chmp4b|LOC100359642 | Chromatin modifying protein 4B-like 1|chromatin modifying protein 4B|rCG37275-like | 1.550 |
| 680047 | Pcdhb9 | Protocadherin beta 9 | 1.482 |
| 24163 | Acr | Acrosin | 1.469 |
| 288596 | Trim50 | Tripartite motif- containing 50 | 1.415 |
| 361749 | Il33 | Interleukin 33 | 1.384 |
| 313954 | Matn3 | Matrilin 3 | 1.357 |
| 100366209 | LOC100366209 | Endothelin-1 receptor-like | 1.336 |
| 360457 | Figf | c-fos induced growth factor | 1.314 |
| 252897 | Pcdhga11 | Protocadherin gamma subfamily A, 11 | 1.292 |
| 317268 | Arx | Aristaless related homeobox | 1.248 |
| 291936|100366089 | Hhip|LOC100366089 | Hedgehog-interacting protein|Hedgehog-interacting protein-like | 1.239 |
| 25250 | Cox8b | Cytochrome c oxidase, subunit VIIIb | 1.224 |
| 289424 | Xpr1 | Xenotropic and polytropic retrovirus receptor 1 | 1.207 |
| 116600 | Cacnb2 | Calcium channel, voltage-dependent, beta 2 subunit | 1.203 |
| 691083 | LOC691083 | Hypothetical protein LOC691083 | 1.201 |
| 192360 | Eml2 | Echinoderm microtubule associated protein like 2 | 1.190 |
| 83792 | Scd | Stearoyl-CoA desaturase (delta-9-desaturase) | 1.185 |
| 499766 | Ttf1 | Transcription termination factor, RNA polymerase I | 1.184 |
| 404796 | Olr778 | Olfactory receptor 778 | 1.176 |
| 25107 | Avpr1a | Arginine vasopressin receptor 1A | 1.162 |
| 79438 | Igfals | Insulin-like growth factor binding protein, acid labile subunit | 1.162 |
| 307212 | Cndp1 | Carnosine dipeptidase 1 (metallopeptidase M20 family) | 1.153 |
| 503446 | Asb12 | Ankyrin repeat and SOCS box-containing 12 | 1.139 |
| 303480 | Hoxb13 | Homeo box B13 | 1.137 |
| 299903 | Slc30a8 | Solute carrier family 30 (zinc transporter), member 8 | 1.130 |
| 291388 | Cbln2 | Cerebellin 2 precursor | 1.122 |
| 252858 | Frmd4b | FERM domain containing 4B | 1.120 |
| 313954 | Matn3 | Matrilin 3 | 1.116 |
| 315163 | Shisa8 | Shisa homolog 8 (Xenopus laevis) | 1.110 |
| 685823 | Xpnpep3 | X-prolyl aminopeptidase (aminopeptidase P) 3, putative | 1.109 |
| 303631 | Arsg | Arylsulfatase G | 1.109 |
| 363849 | Hira | HIR histone cell cycle regulation defective homolog A (S. cerevisiae) | 1.092 |
| 686841 | LOC686841 | Similar to Protein EAN57 | 1.085 |
| 502389 | Npm3 | Nucleophosmin/nucleoplasmin, 3 | 1.077 |
| 306451 | Odz3 | Odz, odd Oz/ten-m homolog 3 (Drosophila) | 1.072 |
| 405936 | Olr445 | Olfactory receptor 445 | 1.061 |
| 311399 | Usp50 | Ubiquitin specific peptidase 50 | 1.053 |
| 309613 | Ng35 | Ng35 pseudogene | 1.036 |
| 361787|414788 | RT1-T24-1|RT1-T24-3 | RT1 class I, locus T24, gene 1|RT1 class I, locus T24, gene 3 | 1.032 |
| 56266 | Cyp4f1 | Cytochrome P450, family 4, subfamily f, polypeptide 1 | 1.029 |
| 140927 | Selenbp1 | Selenium binding protein 1 | 1.025 |
| 306091 | Pcdh9 | Protocadherin 9 | 1.025 |
| 25746 | Il2rb | Interleukin 2 receptor, beta | 1.02 |
| 170928 | Necab2 | N-terminal EF-hand calcium binding protein 2 | 1.014 |
| 29237 | Penk | Proenkephalin | 1.006 |
| 293180 | Micalcl | MICAL C-terminal like | 1.002 |

| **Gene ID** | **Gene symbol** | **Gene description** | **Fold change** |
| --- | --- | --- | --- |
| 171562 | Ero1l | ERO1-like (S. cerevisiae) | -1.794 |
| 361945 | Postn | Periostin, osteoblast specific factor | -1.680 |
| 362505 | Ccl27 | Chemokine (C-C motif) ligand 27 | -1.605 |
| 117505 | Csrp3 | Cysteine and glycine-rich protein 3 (cardiac LIM protein) | -1.584 |
| 302642 | Sat1 | Spermidine/spermine N1-acetyl transferase 1 | -1.519 |
| 170580 | Fgf21 | Fibroblast growth factor 21 | -1.483 |
| 79243 | Hsd17b2 | Hydroxysteroid (17-beta) dehydrogenase 2 | -1.468 |
| NA | NA | NA | -1.435 |
| 29433 | Pak3 | p21 protein (Cdc42/Rac)-activated kinase 3 | -1.426 |
| 684871 | LOC684871 | Similar to Protein C8orf4 (Thyroid cancer protein 1) (TC-1) | -1.420 |
| 24315 | Prl8a2 | Prolactin family 8, subfamily a, member 2 | -1.400 |
| 64440 | Syt4 | Synaptotagmin IV | -1.393 |
| 363227 | Obfc2a | Oligonucleotide/oligosaccharide-binding fold containing 2A | -1.362 |
| 307305 | Prdm6 | PR domain containing 6 | -1.354 |
| 305083 | Zfp281 | Zinc finger protein 281 | -1.338 |
| 365652 | Col4a3bp | Collagen, type IV, alpha 3 (Goodpasture antigen) binding protein | -1.333 |
| 85426 | Slc5a7 | Solute carrier family 5 (choline transporter), member 7 | -1.329 |
| 296787 | Sema3c | Sema domain, immunoglobulin domain (Ig), short basic domain, secreted, (semaphorin) 3C | -1.320 |
| 304832 | Cdc73 | Cell division cycle 73, Paf1/RNA polymerase II complex component, homolog (S. cerevisiae) | -1.315 |
| NA | NA | NA | -1.307 |
| 303039 | Wwc1 | WW and C2 domain containing 1 | -1.303 |
| 313834 | Prkd3 | Protein kinase D3 | -1.295 |
| 361029 | Ktn1 | Kinectin 1 | -1.292 |
| 364031 | Tnfsf18 | Tumor necrosis factor (ligand) superfamily, member 18 | -1.287 |
| 117061 | Mmp10 | Matrix metallopeptidase 10 | -1.286 |
| 24770 | Ccl2 | Chemokine (C-C motif) ligand 2 | -1.284 |
| 300242 | Krt7 | Keratin 7 | -1.282 |
| 360647 | Icam2 | Intercellular adhesion molecule 2 | -1.281 |
| 155140 | Il23a | Interleukin 23, alpha subunit p19 | -1.276 |
| 497865 | RGD1565486 | Similar to RNA binding motif protein 25 | -1.271 |
| 362484 | Plekhf2 | Pleckstrin homology domain containing, family F (with FYVE domain) member 2 | -1.264 |
| 113976 | Acsl4 | Acyl-CoA synthetase long-chain family member 4 | -1.254 |
| 29721 | Hivep2 | Human immunodeficiency virus type I enhancer binding protein 2 | -1.244 |
| 266808 | Lrrcc1 | Leucine rich repeat and coiled-coil domain containing 1 | -1.227 |
| 361029 | Ktn1 | Kinectin 1 | -1.227 |
| 24617 | Serpine1 | Serpin peptidase inhibitor, clade E (nexin, plasminogen activator inhibitor type 1), member 1 | -1.225 |
| 304988 | Ifi204 | Interferon activated gene 204 | -1.202 |
| 60325 | Serpinb2 | Serpin peptidase inhibitor, clade B (ovalbumin), member 2 | -1.192 |
| 100363987 | LOC100363987 | mCG114897-like | -1.189 |
| 304071 | Sim2 | Single-minded homolog 2 (Drosophila) | -1.188 |
| 290577 | LOC290577 | Hypothetical LOC290577 | -1.179 |
| NA | NA | NA | -1.173 |
| 298247 | Mysm1 | Myb-like, SWIRM and MPN domains 1 | -1.169 |
| 500244 | Aak1 | AP2 associated kinase 1 | -1.166 |
| 65054 | Aqp9 | Aquaporin 9 | -1.163 |
| 499660 | Sprr1a | Small proline-rich protein 1A | -1.162 |
| 50555 | Ugt8 | UDP glycosyltransferase 8 | -1.161 |
| 29616 | Ptprm | Protein tyrosine phosphatase, receptor type, M | -1.160 |
| 288515 | Micall2 | MICAL-like 2 | -1.148 |
| 688401|100364099 | Crct1|LOC100364099 | Cysteine-rich C-terminal 1|hypothetical protein LOC100364099 | -1.143 |
| 171516 | Akr1c3 | Aldo-keto reductase family 1, member C3 (3-alpha hydroxysteroid dehydrogenase, type II) | -1.140 |
| 314126 | Baz1a | Bromodomain adjacent to zinc finger domain, 1A | -1.132 |
| 679906 | Trpm7 | Transient receptor potential cation channel, subfamily M, member 7 | -1.131 |
| 85246 | Gas7 | Growth arrest specific 7 | -1.126 |
| 29616 | Ptprm | Protein tyrosine phosphatase, receptor type, M | -1.126 |
| 499558 | March11 | Membrane-associated ring finger (C3HC4) 11 | -1.125 |
| 295647 | Gca | grancalcin | -1.118 |
| 363458 | LOC363458 | Similar to procollagen, type IV, alpha 6 | -1.108 |
| 117033 | Mmp12 | matrix metallopeptidase 12 | -1.107 |
| 29461 | Vgf | VGF nerve growth factor inducible | -1.107 |
| 29611 | Dbt | Dihydrolipoamide branched chain transacylase E2 | -1.107 |
| 64033 | Ccnd2 | Cyclin D2 | -1.106 |
| 690528 | Pou2af1 | POU class 2 associating factor 1 | -1.102 |
| 81783|680385 | Ssb|LOC680385 | Sjogren syndrome antigen B|similar to Sjogren syndrome antigen B | -1.095 |
| 83626 | Ugcg | UDP-glucose ceramide glucosyltransferase | -1.091 |
| 499663 | Lce1f | Llate cornified envelope 1F | -1.088 |
| 498209 | Serpinb3a | Serine (or cysteine) peptidase inhibitor, clade B (ovalbumin), member 3A | -1.086 |
| 307098 | Net1 | Neuroepithelial cell transforming 1 | -1.080 |
| 361084 | Lmo7 | LIM domain 7 | -1.077 |
| 54320 | Pdpn | Podoplanin | -1.077 |
| 84586 | Fgl2 | Fibrinogen-like 2 | -1.069 |
| 64826 | Foxq1 | Forkhead box Q1 | -1.068 |
| 25031 | Plcb4 | Phospholipase C, beta 4 | -1.067 |
| 360600 | Spag9 | Sperm associated antigen 9 | -1.066 |
| 682507 | Wasl | Wiskott-Aldrich syndrome-like | -1.061 |
| 100360606 | LOC100360606 | Zinc finger, RAN-binding domain containing 1 protein-like | -1.059 |
| 25026 | Adm | Adrenomedullin | -1.055 |
| 362521 | Tmem38b | Transmembrane protein 38B | -1.048 |
| 299012 | Arhgap5 | Rho GTPase activating protein 5 | -1.047 |
| 78963 | Apaf1 | Apoptotic peptidase activating factor 1 | -1.046 |
| 288227 | Bace2 | Beta-site APP-cleaving enzyme 2 | -1.045 |
| 304266 | Gbp1 | Guanylate binding protein 1, interferon-inducible | -1.044 |
| 295193 | RGD1308305 | Similar to RIKEN cDNA 5430400H23 | -1.042 |
| 313874 | Rasgrp3 | RAS guanyl releasing protein 3 (calcium and DAG-regulated) | -1.038 |
| NA | NA | NA | -1.037 |
| NA | NA | NA | -1.036 |
| 307479 | Jakmip2 | Janus kinase and microtubule interacting protein 2 | -1.033 |
| 499797 | RGD1560248 | Similar to formin-like 2 isoform B | -1.032 |
| 307562 | Dsg2 | Desmoglein 2 | -1.031 |
| 192359 | Zc3h14 | Zinc finger CCCH type containing 14 | -1.027 |
| 365894 | Trim33 | Tripartite motif-containing 33 | -1.021 |
| 680477 | Haus3 | HAUS augmin-like complex, subunit 3 | -1.019 |
| 294151 | Olr1684 | Olfactory receptor 1684 | -1.017 |
| 25353 | Spp1 | Secreted phosphoprotein 1 | -1.010 |
| 84481 | Arid4b | AT rich interactive domain 4B (Rbp1 like) | -1.008 |
| 306628 | Col4a2 | Collagen, type IV, alpha 2 | -1.007 |
| 50552 | Zfp292 | Zinc finger protein 292 | -1.006 |
| 619374 | Jam2 | Junctional adhesion molecule 2 | -1.003 |

1. **Up-regulated ( ≥ 2.0-fold) and down-regulated ( ≤ 2.0-fold) genes in PC12 cells after Aβ_25-35_ injury as compared with control group.**

| **Gene ID** | **Gene symbol** | **Gene description** | **Fold change** |
| --- | --- | --- | --- |
| 688915 | Cmya5 | Cardiomyopathy associated 5 | 3.613 |
| 305501 | Ikzf1 | IKAROS family zinc finger 1 | 2.417 |
| 315980 | RGD1307220 | Similar to RIKEN cDNA E330026B02 | 1.167 |
| 500337 | Clec1a | C-type lectin domain family 1, member A | 1.084 |
| 89823 | Trpc6 | Transient receptor potential cation channel, subfamily C, member 6 | 1.057 |

| **Gene ID** | **Gene symbol** | **Gene description** | **Fold change** |
| --- | --- | --- | --- |
| 192266|360395 | Grpca|Grpcb | Glutamine/glutamic acid-rich protein A |glutamine/glutamic acid-rich protein A | -1.829 |
| NA | NA | NA | -1.660 |
| 361945 | Postn | Periostin, osteoblast specific factor | -1.388 |
| 25109 | Cd1d1 | CD1d1 molecule | -1.307 |
| NA | NA | NA | -1.294 |
| 84029 | Hao2 | Hydroxyacid oxidase 2 (long chain) | -1.215 |
| 64543 | Sec14l3 | SEC14-like 3 (S. cerevisiae) | -1.199 |
| 362368 | Ggct | Gamma-glutamyl cyclotransferase | -1.157 |
| 362378 | Fam13a | Family with sequence similarity 13, member A | -1.091 |
| 502731 | Vwde | Von Willebrand factor D and EGF domains | -1.086 |
| 246358 | Ppbp | Pro-platelet basic protein (chemokine (C-X-C motif) ligand 7) | -1.076 |
| 100158232 | Zfp9 | Zinc finger protein 9 | -1.066 |
| 100359536 | LOC100359536 | Spermatogenesis associated glutamate (E)-rich protein 4e-like | -1.042 |
| 288596 | Trim50 | Tripartite motif- containing 50 | -1.023 |
| 192266|360395 | Grpca|Grpcb | Glutamine/glutamic acid-rich protein A |glutamine/glutamic acid-rich protein A | -1.022 |
| 691767 | Vom2r34 | Vomeronasal 2 receptor, 34 | -1.003 |
